# Supplementary material for: Evidence for dynamic kagome ice
Source: Nat Commun. 2018 Sep 17;9:3786. doi: 10.1038/s41467-018-06212-2 (PMC6141606; doi:10.1038/s41467-018-06212-2)
Supplement: Supplementary file 1 — Supplementary Information [file 41467_2018_6212_MOESM1_ESM.pdf]

Evidence for dynamic kagome ice
Supplementary Information

E. Lhotel et al.

Site	1	2	3	4
CEF axis $\mathbf{z_i}$	$(1,1,\bar{1})$	$(\bar{1},\bar{1},\bar{1})$	$(\bar{1},1,1)$	$(1,\bar{1},1)$
Coordinates	$(1/4,1/4,1/2)$	$(0,0,1/2)$	$(0,1/4,3/4)$	$(1/4,0,3/4)$
$\mathbf{a_i}$	$(\bar{1},\bar{1},\bar{2})$	$(1,1,\bar{2})$	$(1,\bar{1},2)$	$(\bar{1},1,2)$
$\mathbf{b_i}$	$(\bar{1},1,0)$	$(1,\bar{1},0)$	$(1,1,0)$	$(\bar{1},\bar{1},0)$

Supplementary Table 1. **Site coordinates**. They are written in the cubic $Fd\bar{3}m$ structure of the pyrochlore lattice, for the site dependent local $\mathbf{a_i}$ and $\mathbf{b_i}$ vectors spanning the local $(\mathbf{x},\mathbf{y})$ anisotropy planes. The CEF axes $\mathbf{z_i}$ of the rare earth ions are perpendicular to these planes.

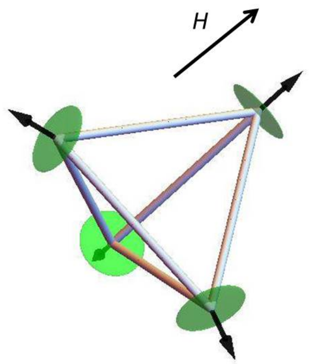

Supplementary Figure 1. **Sketch of a tetrahedron in the pyrochlore lattice.** The black arrows show the local $\mathbf{z}_i$ anisotropy (crystal field - CEF) axes and the green disks represent the local $(\mathbf{a}_i,\mathbf{b}_i)$ planes. Further details are given in Supplementary Table 1.

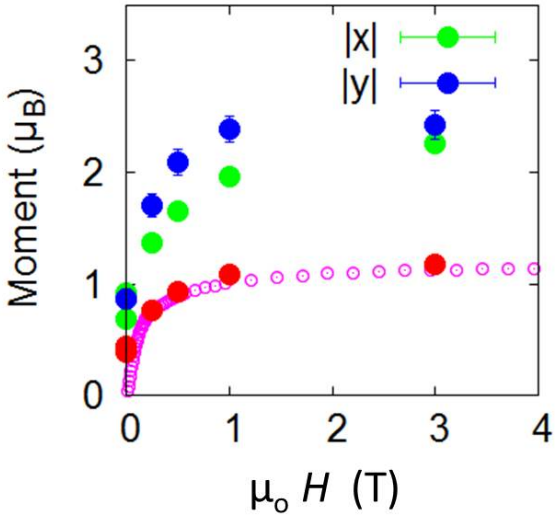

Supplementary Figure 2. **Field dependence of the magnetic moments in the unit cell for H** $\parallel$ [111]**.** y (in blue) gives the moment of the apical spin, with its CEF axis along the field. x (in green) gives the moment of the three remaining spins (also called kagome). The magenta curve displays the magnetization obtained from macroscopic measurements. Red points correspond to the magnetization calculated using the refinements results and projected onto the field direction, leading to $M(H)=(x-y)/4$. The error bars are provided by the Rietveld refinement made using Fullprof.

Supplementary Note 1. Neutron diffraction

Experimental details: The neutron diffraction data were collected using the D23 single crystal diffractometer (CEA-CRG, ILL France) operated with a copper monochromator and using $\lambda = 1.28$ Å. The single crystal of $Nd_2Zr_2O_7$ was glued on the Cu finger of a dilution insert and placed in a cryomagnet. The experiments have been conducted with the (vertical) field $\mathbf{H}$ parallel to the [111] direction. We first checked that the Bragg intensities remain zero on the forbidden $\mathbf{q}$ vectors of the F centred lattice. This implies that the field induced structures are described by a $\mathbf{k} = \mathbf{0}$ propagation vector. The additional magnetic intensity is then observed on top of the nuclear Bragg peaks. A series of integrated intensities at 10 K and $H = 0$ was measured (about 60 reflections) as a reference. Refinements were performed using the FULLPROF sofware suite[1], working on the differences with the $T = 10$ K data set. Finally, for a set of chosen Bragg peaks, we ramped the field back and forth, up to 1 and back to -1 T, yielding a precise evolution of the magnetic intensities. The 1 T field was chosen to saturate the sample.

Model of the magnetic structure in a [111] field: Since the propagation vector is $\mathbf{k} = \mathbf{0}$, the four tetrahedra of the crystalline unit cell host identical arrangements of the spins. Furthermore, because of the strong Ising anisotropy of the Nd^{3+} ions, it is reasonable to consider that the magnetic moments are forced to align along the local CEF axis (labelled $\mathbf{z}_i$, see Supplementary Figure 1 and Table 1). In our refinement, we thus assume the following model (see Table 1):

$$\begin{aligned} \mathbf{m}_2 &= y\ \mathbf{z}_2 \\ \mathbf{m}_i &= x\ \mathbf{z}_i, \quad i = 1,3,4 \end{aligned} \tag{1}$$

where x and y are the fitted parameters. Note that $\mathbf{m}_2$ is parallel to [111], hence to the applied field. From a physical point of view, situations where x and y have the same sign correspond to a generalized all in – all out structure. When x and y have different signs, however, the model describes a structure which resembles the 1 out – 3 in, but $\mathbf{m}_2$ and $\mathbf{m}_{1,3,4}$ may have different amplitudes. The magnetization per Nd^{3+} ion is given by:

$$\mathbf{M} = \frac{1}{4} \sum_{i \in \boxtimes = \{1,2,3,4\}} \mathbf{m}_i = \frac{(x-y)}{4\sqrt{3}} \begin{pmatrix} 1 \\ 1 \\ 1 \end{pmatrix} \tag{2}$$

Projected along the [111] field, it simply becomes $M = (x - y)/4$. The results obtained increasing the field from 0 to 3 T along with the calculated magnetization are shown in Supplementary Figure 2. The excellent agreement with the macroscopic magnetization is also shown for comparison. It is worth noting that the magnetic structure may also be described in terms of generalized charges $\mathbf{Q}_\boxtimes$ living on the dual (diamond) lattice defined by the centres of the tetrahedra. $\mathbf{Q}_\boxtimes$ is a vector with three components defined by:

$$\begin{aligned} Q^x_\boxtimes &= \sum_{i \in \boxtimes = \{1,2,3,4\}} \mathbf{m}_i.\mathbf{a}_i \\ Q^y_\boxtimes &= \sum_{i \in \boxtimes = \{1,2,3,4\}} \mathbf{m}_i.\mathbf{b}_i \\ Q^z_\boxtimes &= \sum_{i \in \boxtimes = \{1,2,3,4\}} \mathbf{m}_i.\mathbf{z}_i = 3x + y \end{aligned} \tag{3}$$

In zero field, the all in – all out structure can be considered as a Q^z charged staggered pattern.

Analysis of the ramps: We now turn to the analysis of the intensities measured while ramping the magnetic field (see Supplementary Figure 3). To this end, we first write the magnetic structure factor for a wavevector $\mathbf{q}$:

$$\mathbf{F}_\mathrm{M}(\mathbf{q}) = \sum_i \mathbf{m}_i\ f_i\ e^{i\mathbf{q}\mathbf{R}_i} \tag{4}$$

where $\mathbf{R}_i$ denotes the position of the ith spin in the unit cell and f_i is the form factor. The magnetic Bragg intensity is then given by: $I_\mathbf{q} = \sum_{a,b=x,y,z} \mathbf{F}^a_\mathrm{M}(\mathbf{q}) \left(\delta_{a,b} - \frac{q^a q^b}{q^2}\right) \mathbf{F}^b_\mathrm{M}(\mathbf{q})^*$. For $\mathbf{q} = (2,0,0)$, $(1,\bar{1},1)$ and $(1,3,\bar{1})$, analytic

calculations show that:

$$\begin{aligned} I_{200} - I^{\rm N}_{200} &= 32V(x-y)^2 \\ I_{1\bar{1}1} - I^{\rm N}_{1\bar{1}1} &= 42.66V(x-y)^2 \\ I_{13\bar{1}} - I^{\rm N}_{13\bar{1}} &= V\left(Ax^2 + 2Bxy + Cy^2\right) \end{aligned} \quad (5)$$

where V is the volume, $I^{\rm N}_{\mathbf{q}}$ denote the corresponding nuclear contributions and the coefficients are given by: $A = 174.55$, $B = -11.64$ and $C = 34.91$. In these expressions, the form factor has been neglected since it is close to 1 for those $\mathbf{q}$ vectors. It is then convenient to consider the new intermediate variables e and t defined as:

$$\begin{aligned} e = &\quad y - x \\ t = &\quad y/x - 1 \\ \Rightarrow &\quad e = tx \end{aligned} \quad (6)$$

hence:

$$\begin{aligned} & I_{200} - I^{\rm N}_{200} = 32Ve^2 \\ & I_{1\bar{1}1} - I^{\rm N}_{1\bar{1}1} = 42.66Ve^2 \\ & I_{13\bar{1}} - I^{\rm N}_{13\bar{1}} = V\left((A+2B+C) + 2(B+C)t + Ct^2\right)\frac{e^2}{t^2} \qquad \text{when } t, e \neq 0. \end{aligned} \quad (7)$$

Note that $e = 0$ at 0 T since the structure is all in – all out ($x = y$). We now consider reduced quantities where the o and 1 superscripts denote respectively the values at 0 and 1 T. We then have $I^{\rm o}_{1\bar{1}1} = I^{\rm N}_{1\bar{1}1}$ and $I^{\rm o}_{200} = I^{\rm N}_{200}$. By introducing $\zeta = I^{\rm o}_{13\bar{1}} - I^{\rm N}_{13\bar{1}} = V\,(A+2B+C)\,x_o^2$, $\mu = 1/e_1^2$ and $\eta = I^1_{13\bar{1}} - I^{\rm o}_{13\bar{1}}$,

$$\begin{aligned} i_{200} &= \frac{I_{200} - I^{\rm o}_{200}}{I^1_{200} - I^{\rm o}_{200}} = \frac{e^2}{e_1^2} = \mu e^2 \\ i_{1\bar{1}1} &= \frac{I_{1\bar{1}1} - I^{\rm o}_{1\bar{1}1}}{I^1_{1\bar{1}1} - I^{\rm o}_{1\bar{1}1}} = \frac{e^2}{e_1^2} = \mu e^2 \\ i_{13\bar{1}} &= \frac{I_{13\bar{1}} - I^{\rm o}_{13\bar{1}}}{I^1_{13\bar{1}} - I^{\rm o}_{13\bar{1}}} \\ &= \eta\left[\frac{e^2}{t^2}\left((A+2B+C) + 2(B+C)t + Ct^2\right) - \zeta\right]. \end{aligned} \quad (8)$$

As a result, the variable e is readily obtained from the field evolution of the $(2,0,0)$ or $(1,\bar{1},1)$ intensity:

$$e^2 = i_{200}/\mu. \quad (9)$$

Note that the data are consistent, i.e. i_{200} and $i_{1\bar{1}1}$ give the same information. Finally, we need to solve the following equation, deduced from the expression for $i_{13\bar{1}}$:

$$0 = t^2\left(1 - \frac{Ce^2}{i_{13\bar{1}}/\eta + \zeta}\right) - 2t(B+C)\frac{e^2}{i_{13\bar{1}}/\eta + \zeta} - (A+2B+C)\frac{e^2}{i_{13\bar{1}}/\eta + \zeta}. \quad (10)$$

For each field value, or in other words, for each e value implicitly determined from i_{200}, there exist two solutions t_1 and t_2, hence two solutions for the $y/x = (1+t)$ ratio. These solutions take the form of the black and grey branches shown in Supplementary Figure 4a. The final choice between the two possibilities relies on physical grounds and is shown by the red points in Supplementary Figure 4a: when the field is strongly negative or positive, a 3 in – 1 out structure is expected, i.e. $y/x \leq 0$. This corresponds to the black branch in Supplementary Figure 4a. In zero field, the all in – all out structure is stabilized, which corresponds to $y/x = 1$, hence to the point where the black and grey solutions coincide in Supplementary Figure 4b. Since x and y should be regular functions of the field, except close to the transition at 0.08 T where a discontinuity can be expected, the physical solution stays on the black branch. This holds up to the transition at 0.08 T. Above, there is a jump to join the grey solution. This gives the field evolution for the x and y parameters shown in Supplementary Figure 4c. The blue arrow marks the discontinuity at the transition.

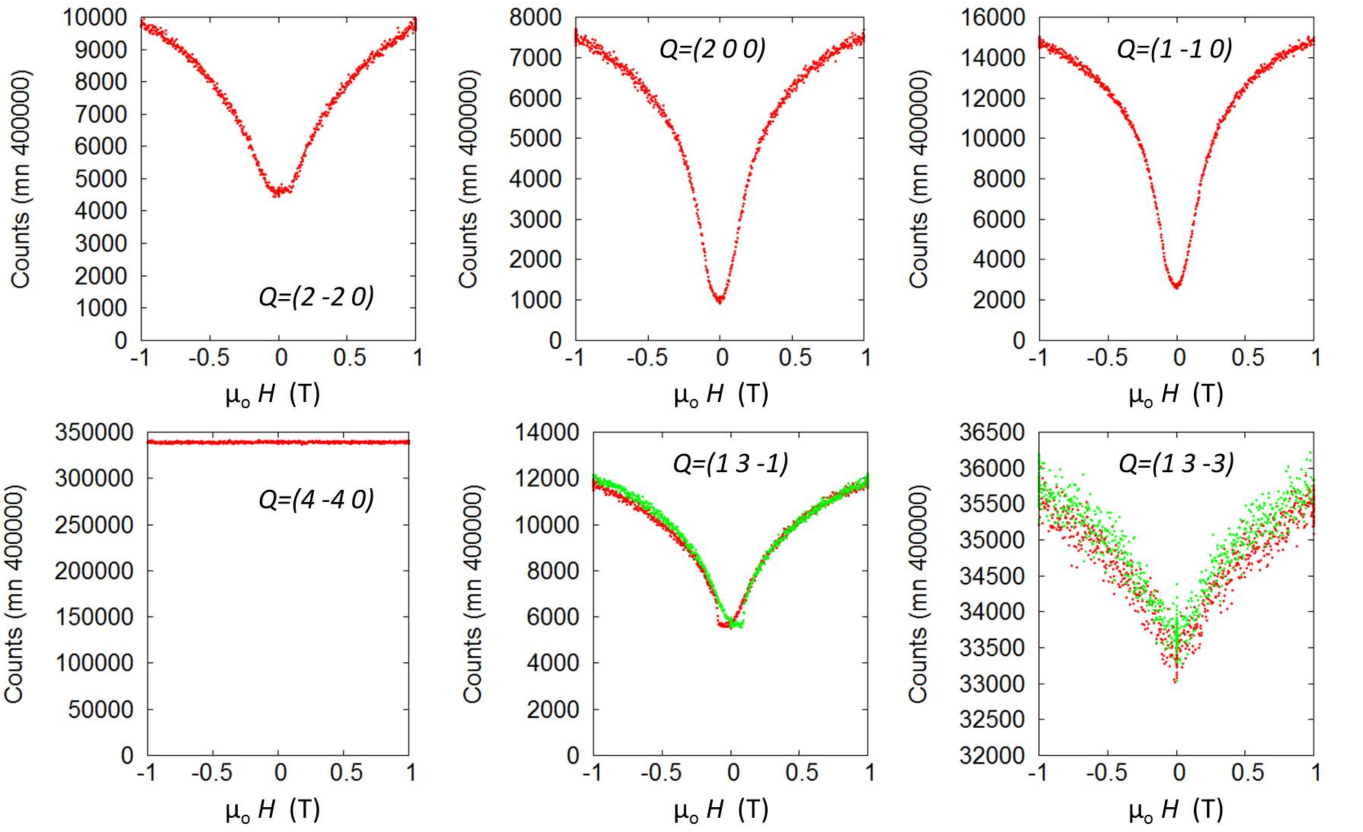

Supplementary Figure 3. **Bragg peak intensities vs** H**, with the field applied along** [111]. They were measured while ramping the field back and forth up to 1 and back to -1 T. Red and green points correspond to sweeping the field from positive and negative values respectively.

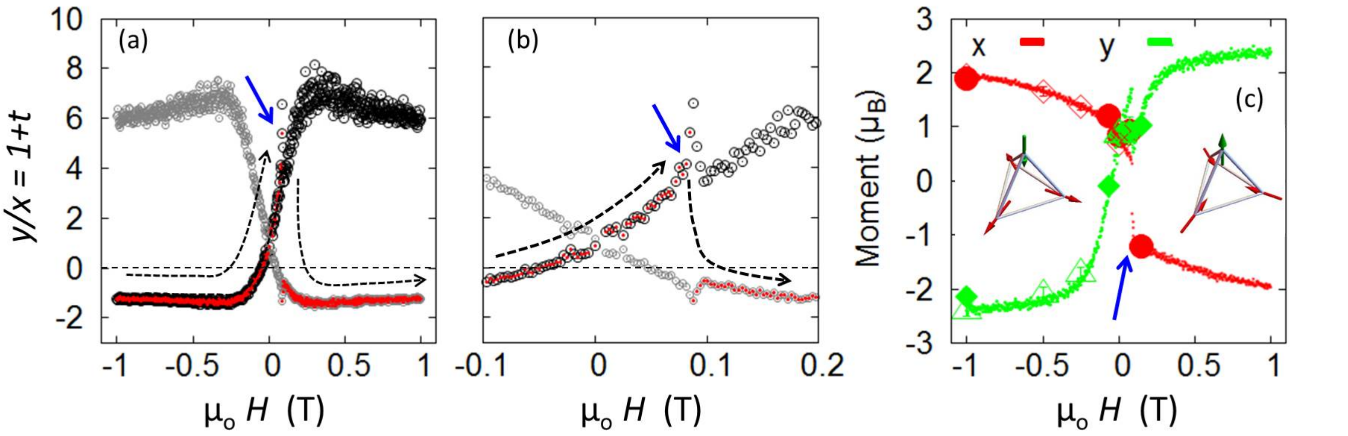

Supplementary Figure 4. **Field dependence of the variables defined in Supplementary equation 6.** Figures (a) and (b) show $y/x = (1+t)$ as a function of the field. Two branches (grey and black) are found. The blue arrow marks the transition and the dotted black arrows the choice between the two branches. (c) shows the field dependence of x and y (closed symbols). As a complement, data were taken with a graphite PG monochromator, reported here with open symbols. Note the satisfying agreement with the Fullprof refinement.

Supplementary Note 2. Spin dynamics and inelastic neutron scattering

Inelastic neutron scattering experiments were carried out on the IN5 disk chopper time of flight spectrometer operated by the Institute Laue Langevin (ILL, France). The field was applied along [111] and the $Nd_2Zr_2O_7$ single crystal mounted to have the $(-k, k, 0)$-$(-h, -h, 2h)$ reciprocal directions in the horizontal scattering plane. A sketch of the $(-k, k, 0)$-$(-h, -h, 2h)$ scattering plane along with the positions of remarkable wavevectors is displayed in Supplementary Figure 5. The kagome ice pinch points (in yellow) and all in – all out positions (in blue) are especially shown.

As a very good energy resolution is needed (about 20 μeV), we used a wavelength $\lambda = 8.5$ Å. The data were then processed with the HORACE software[2], transforming the recorded time of flight, sample rotation and scattering angle into energy transfer and q-wave-vectors. The offset of the sample rotation was determined based on the Bragg peak positions. In all the experiments, the sample was rotated in steps of 1 degree. We finally prepared a set of constant $\mathbf{q}$ scans by integrating over a small $\mathbf{q}$ range with $\Delta h = \Delta k = 0.05$. We also prepared a series of constant energy maps (ranging from $E = 0.05$ up to 0.25 meV) that were symmetrized on the basis of the expected 6-fold symmetry of the intensity in this scattering plane. These maps are displayed in Supplementary Figure 7 and 8, giving an overview of the spin dynamics for a series of applied magnetic fields. Note that those maps were obtained by integrating over a range $\Delta\omega = 10$ μeV, which is about half the energy resolution. Supplementary Figure 6 shows the corresponding $\mathbf{q}$-integrated spectra. Finally, Supplementary Figure 9 and 10 display the dispersions measured along the black dotted and full slices shown in Supplementary Figure 5.

Supplementary Figures 6 to 10 show that there is little evolution of the scattering at low field, i.e. for $\mu_0 H = 0$, 0.07 and 0.15 T. A dispersive branch (marked by the A symbol) is identified in the Supplementary Figures 9 and 10. While the top of this branch clearly appears at $(-2, 2, 0)$ and symmetry related points (with an energy of 0.25 meV), it is difficult to determine from these dispersions, whether or not this branch reaches a minimum. A close look at Supplementary Figure 7 shows that the branch does reach a minimum at the $(-4/3, 2/3, 2/3)$-like points, with an energy of about 0.1 meV, thus above the energy of the flat mode. This can be observed in the two left columns of Supplementary Figures 7 and 8, and is marked by the A symbol on Supplementary Figures 9 and 10.

Above $\mu_0 H = 0.25$ T, a different picture sets in. First of all, the structure factor of the flat mode evolves: the intensity close to $\mathbf{q} = 0$ strongly decreases while 6 arms take shape. With increasing field, the intensity of the arms strongly decreases (see the first row of Supplementary Figure 7; note also the change in the color-scale). In addition, two narrow and dispersive branches, labelled A and B, can be observed (see Supplementary Figures 9 and 10). The A branch is descended from the zero field dispersive branch. For larger fields, this branch is still there but is quite difficult to observe since it becomes extremely weak. However, by taking the $\mathbf{q}$-average of the scattering (see Supplementary Figure 6), which basically gives the density of states (enhancing flat modes), it becomes clearly visible. From this data, we can conclude that the A branch bandwidth becomes narrower, and progressively shifts towards higher energies.

The second dispersing B branch emerges from the kagome ice pinch point positions ($(-4/3, 2/3, 2/3)$-like points) above the 6 arms mode and closes at $(-2, 2, 0)$ (and symmetry related points). With increasing the field, this B branch develops up to about 0.10 meV at $\mu_0 H = 0.25$ T. The top of the band slightly shifts to higher energies with further increasing the energy. At $\mu_0 H = 0.75$ T, for instance, it shifts up to 0.13 meV.

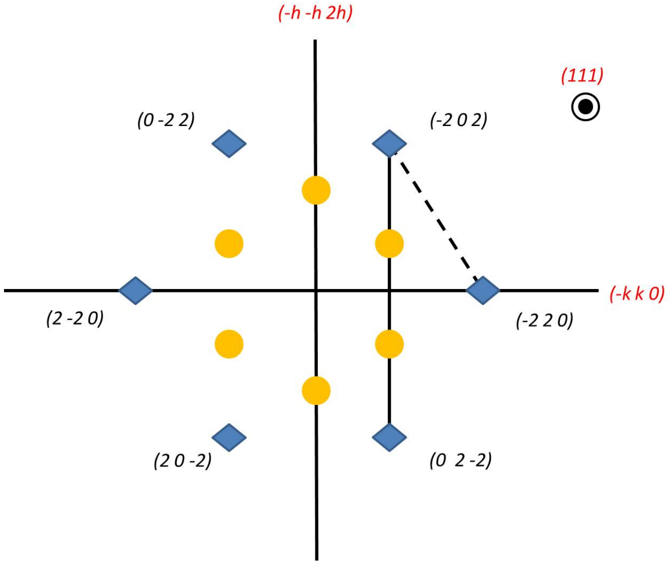

Supplementary Figure 5. **Sketch of the** $(-k, k, 0)$ $(-h, -h, 2h)$ **scattering plane for a** [111] **vertical magnetic field axis**. The blue diamonds correspond to the all in – all out Bragg peak positions observed in zero field. The yellow points show the positions of the pinch points arising in the kagome ice model. These positions are of the form $(-4/3, 2/3, 2/3)$ and symmetry related positions. Black dotted line and the full line show the direction of the maps shown in Supplementary Figures 9 and 10 respectively.

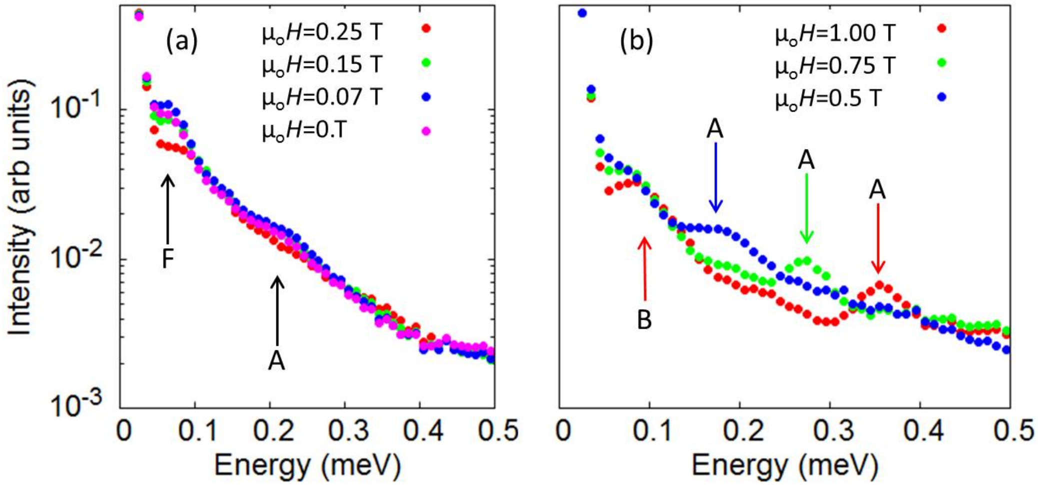

Supplementary Figure 6. q**-integrated spectra for a series of magnetic fields applied along** [111]. In (a), the arrows labelled F and A correspond respectively to the flat mode and to the A dispersive mode described in the text. In (b), the coloured arrows show the position of the A and B branches for different fields. Both branches shift to higher energies with increasing field.

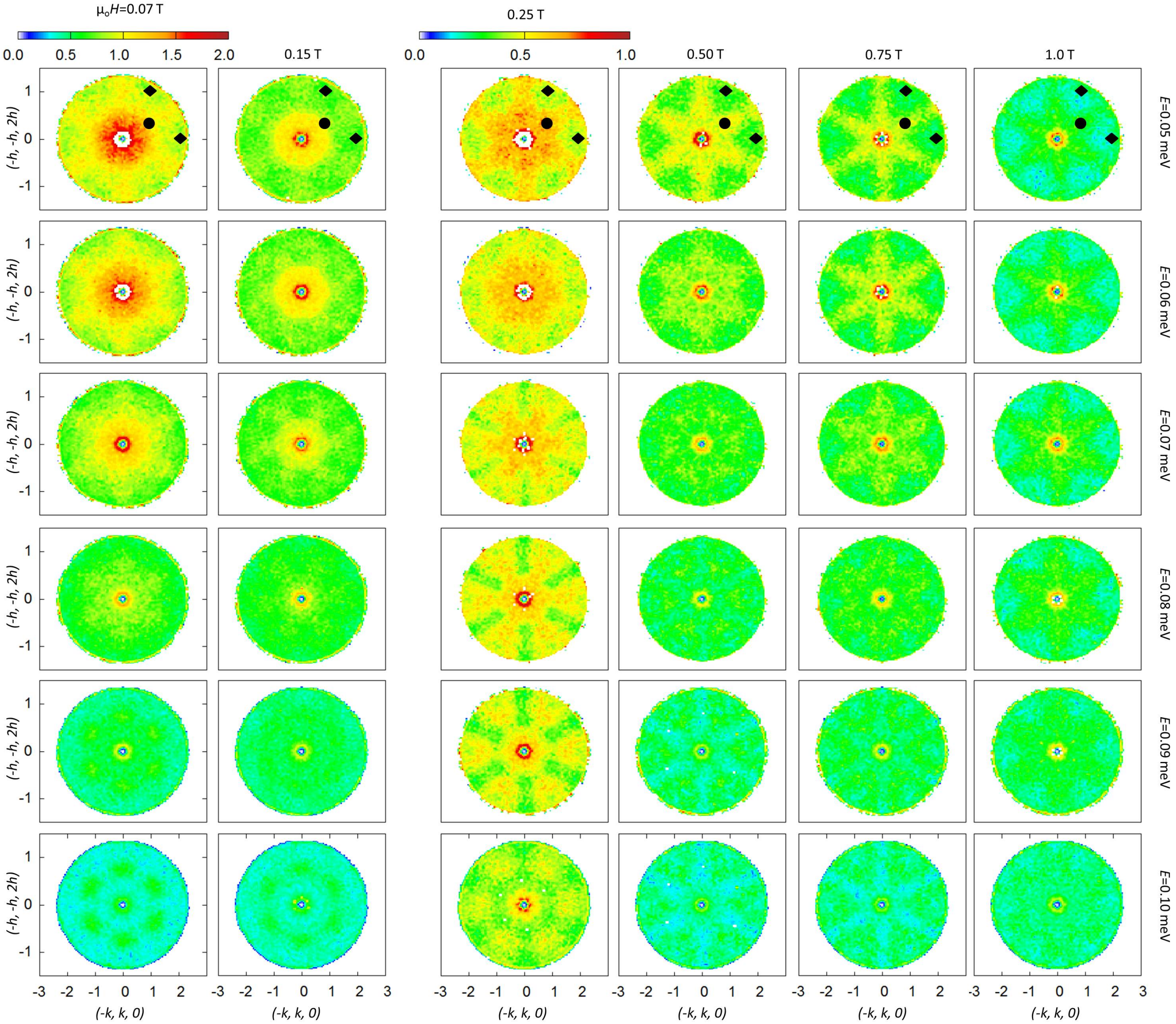

Supplementary Figure 7. Constant energy maps in the $(-k, k, 0)$-$(-h, -h, 2h)$ scattering plane at energies up to 0.1 meV and for fields between 0 and 1 T. They were measured at 60 mK. The black point marks the $(-4/3, 2/3, 2/3)$ position, and the black diamonds mark the $(-2, 2, 0)$ and the $(-2, 0, 2)$ positions.

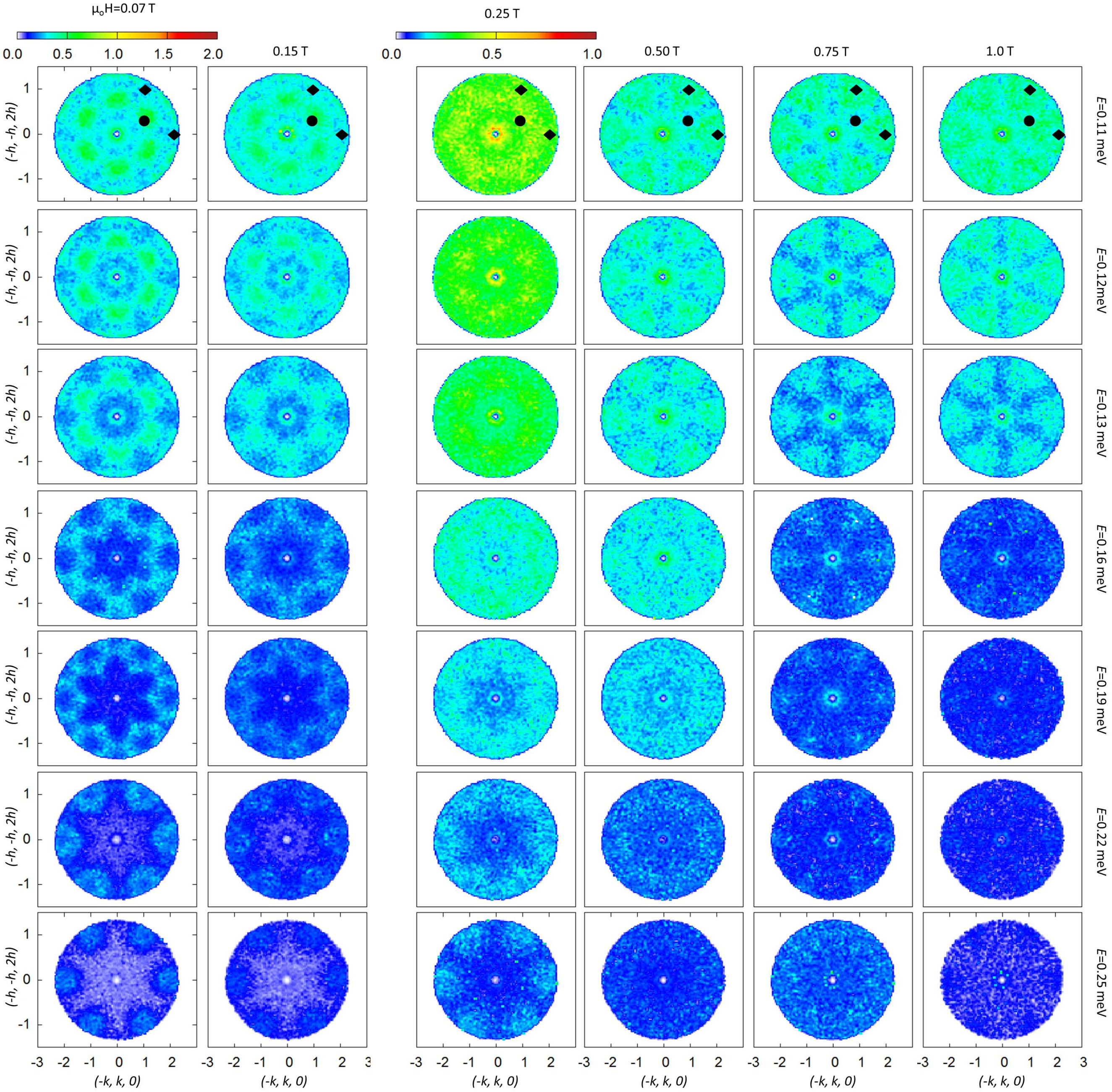

Supplementary Figure 8. **Constant energy maps in the** $(-k, k, 0)$**-**$(-h, -h, 2h)$ **scattering plane at energies between 0.11 and 0.25 meV and for fields between 0 and 1 T**. They were measured at 60 mK. The black point marks the $(-4/3, 2/3, 2/3)$ position, and the black diamonds mark the $(-2, 2, 0)$ and the $(-2, 0, 2)$ positions.

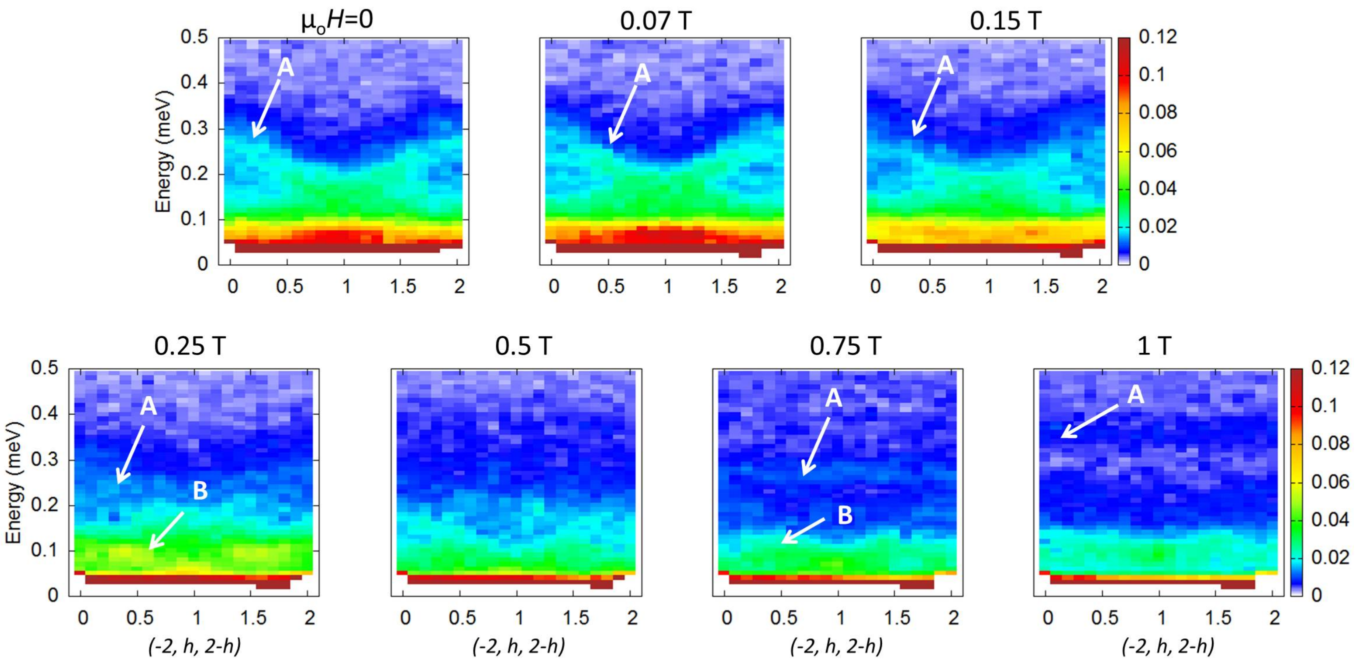

Supplementary Figure 9. **Dispersions along** $(-2, h, 2-h)$ **measured for different fields between 0 and 1 T at 60 mK.** See the black dotted line in Supplementary Figure 5. $\mu_0 H = 0.25$ T is the threshold which clearly separates two regimes.

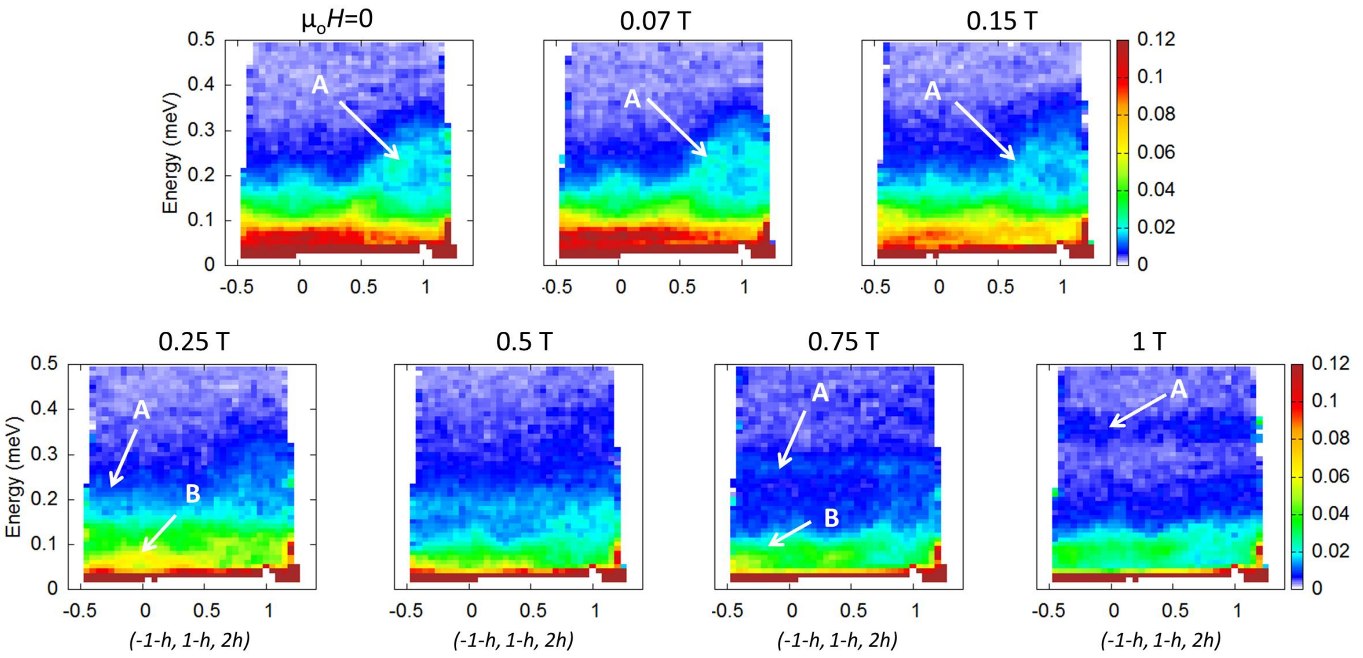

Supplementary Figure 10. **Dispersions along** $(-1-h, 1-h, 2-h)$ **measured for different fields between 0 and 1 T at 60 mK.** See the black line in Supplementary Figure 5.

Supplementary Note 3. The XYZ model

Following literature[3–5], the relevant model to describe the case of $Nd_2Zr_2O_7$ is the XYZ Hamiltonian:

$$\mathcal{H} = \sum_{\langle i,j\rangle} \left(\mathsf{J}_x \tau_i^x \tau_j^x + \mathsf{J}_y \tau_i^y \tau_j^y + \mathsf{J}_z \tau_i^z \tau_j^z + \mathsf{J}_{xz}(\tau_i^x \tau_j^z + \tau_i^z \tau_j^x) \right). \tag{11}$$

In this expression, τ_i is a pseudo-spin that resides on the sites of the pyrochlore lattice. The $(\mathbf{x}, \mathbf{y}, \mathbf{z})$ coordinates refer to the site dependent frames, where $\mathbf{z}$ is the local $\langle 111\rangle$ axis (see also Supplementary Table 1). Note that the $\mathbf{z}$ component is related to the physical magnetic moment $\mathbf{m} = g_{\parallel}\, \tau^z\, \mathbf{z}$, while the $\mathbf{x}$ and $\mathbf{y}$ components are non observable quantities. They respectively transform as dipolar and octupolar moments under symmetries. A rotation by an angle θ in the $(\mathbf{x}, \mathbf{z})$ plane allows one to define a new $(\tilde{\mathbf{x}}, \tilde{\mathbf{z}})$ frame, new pseudo-spin components $(\tilde{\tau}^{\tilde{x}}, \tilde{\tau}^{\tilde{y}}, \tilde{\tau}^{\tilde{z}})$ and new coupling constants $(\tilde{\mathsf{J}}_x, \tilde{\mathsf{J}}_y, \tilde{\mathsf{J}}_z)$ so that the Hamiltonian becomes diagonal:

$$\mathcal{H} = \sum_{\langle i,j\rangle} \left(\tilde{\mathsf{J}}_x \tilde{\tau}_i^{\tilde{x}} \tilde{\tau}_j^{\tilde{x}} + \tilde{\mathsf{J}}_y \tilde{\tau}_i^{\tilde{y}} \tilde{\tau}_j^{\tilde{y}} + \tilde{\mathsf{J}}_z \tilde{\tau}_i^{\tilde{z}} \tilde{\tau}_j^{\tilde{z}} \right). \tag{12}$$

The rotation matrix is defined as:

$$\mathcal{R} = \begin{pmatrix} \cos\theta & & \sin\theta \\ & 1 & \\ -\sin\theta & & \cos\theta \end{pmatrix} \text{ and } \begin{pmatrix} \mathsf{J}_x & & \mathsf{J}_{xz} \\ & \mathsf{J}_y & \\ \mathsf{J}_{xz} & & \mathsf{J}_z \end{pmatrix} = \mathcal{R}^T \begin{pmatrix} \tilde{\mathsf{J}}_x & & \\ & \tilde{\mathsf{J}}_y & \\ & & \tilde{\mathsf{J}}_z \end{pmatrix} \mathcal{R} \tag{13}$$

With this definition, the angle between the $\mathbf{z}$ and $\tilde{\mathbf{z}}$ axes is θ. The following relations are especially useful:

$$\begin{aligned} \tan 2\theta =& \frac{2\mathsf{J}_{xz}}{\mathsf{J}_x - \mathsf{J}_z}, \\ \mathsf{J}_{xz} =& (\tilde{\mathsf{J}}_x - \tilde{\mathsf{J}}_z) \sin\theta \cos\theta, \\ \mathsf{J}_x =& \tilde{\mathsf{J}}_x \cos^2\theta + \tilde{\mathsf{J}}_z \sin^2\theta, \\ \mathsf{J}_z =& \tilde{\mathsf{J}}_z \cos^2\theta + \tilde{\mathsf{J}}_x \sin^2\theta. \end{aligned} \tag{14}$$

Importantly, the energies of the spin wave modes solely depend on the values of the three parameters $\tilde{\mathsf{J}}_x, \tilde{\mathsf{J}}_y$ and $\tilde{\mathsf{J}}_z$. This does not mean that J_{xz} or the angle θ do not play any role; the latter especially appears when projecting the pseudo-spin onto the local $\mathbf{z}$ magnetic direction. As a result, it enters the spin correlation functions (i.e. the inelastic neutron scattering cross section) as well as the magnetization along with its field dependence.

In Supplementary Reference 4, we assumed $\mathsf{J}_x = \mathsf{J}_{xz} = 0$, following arguments based on the analysis of the crystal field. Furthermore, we found that:

$$\mathsf{J}_y = -0.55 \text{ K}, \; \mathsf{J}_z = 1.2 \text{ K} \tag{15}$$

allow us to reproduce quantitatively the INS spectra. These parameters also led to an all in – all out ordering along the y octupolar axis. Note that at the classical level, this particular ordering is stabilized provided that:

$$\mathsf{J}_y \le \mathsf{J}_z \le 3|\mathsf{J}_y| \text{ and } \mathsf{J}_y \le 0. \tag{16}$$

Spin wave calculations showed then that the spectrum encompasses two contributions, a flat mode E_o at:

$$E_o = \sqrt{3|\mathsf{J}_y|(3|\mathsf{J}_y| - \mathsf{J}_z)} \tag{17}$$

along with dispersive branches, in excellent agreement with INS results. It is worth emphasizing that this analysis, however, does not explain the origin of the *magnetic* all in – all out ordering (along the $\mathbf{z}$ axis). It was actually designed in the framework of the fragmentation scenario, following the idea that the magnetization can be decomposed into two independent fragments, the divergence full and the divergence free emergent fields of a Helmholtz-Hodge decomposition. The above description, and especially the condensation of the octupolar ordering, was thus thought to apply to the divergence free part only.

In Supplementary Reference 5, O. Benton followed a different route. Taking advantage of a global symmetry of the

Hamiltonian, he proposed to inter-change the axes compared to Supplementary Reference 4 so that:

$$\tilde{\mathsf{J}}_x = 1.2 \text{ K}, \ \tilde{\mathsf{J}}_y = 0, \ \tilde{\mathsf{J}}_z = -0.55 \text{ K}, \tag{18}$$

and introduced a non zero angle $\theta = 0.83(48^\circ)$ chosen to reproduce the Curie-Weiss temperature. This set of parameters corresponds to:

$$\mathsf{J}_x = 0.247 \text{ K}, \ \mathsf{J}_y = 0, \ \mathsf{J}_z = 0.403 \text{ K}, \ \mathsf{J}_{xz} = 0.871 \text{ K}. \tag{19}$$

For this choice, the classical ground state is an all in – all out configuration with respect to the rotated $\tilde{\mathbf{z}}$ axis. The spins $\langle \tau \rangle$ thus point along the $\tilde{\mathbf{z}}$ axes. This rotated all in – all out order has projections onto the original magnetic $\mathbf{z}$ axis:

$$m = g_{\parallel} \frac{\cos\theta}{2} \tag{20}$$

and along the $\mathbf{x}$ axis:

$$\langle \tau_x \rangle \sim 1/2 \sin\theta \tag{21}$$

(note again that the latter cannot be observed directly with neutrons). INS results are equally well reproduced with these parameters since the spin wave energies do not depend on θ.

Supplementary Note 4. Fitting the INS data

In the following, we adopt the scheme discussed immediately above and in Supplementary Reference 5, but revisit the values of the parameters to fit the data taken in zero and in applied field. Our initial choice of θ is different, and is based on the following relation:

$$\cos\theta = \frac{m^{\text{ord}}}{m^{\text{sat}}} \approx 0.357 \tag{22}$$

where $m^{\text{ord}} = 0.8\mu_{\text{B}}$ is the experimental all in – all out ordered moment and $m^{\text{sat}} = 2.28$ is the calculated saturation moment determined from crystal field coefficients[6]. This corresponds to an angle:

$$\theta \approx 69^\circ. \tag{23}$$

To ensure that an all in – all out state is stabilized along the $\tilde{\mathbf{z}}$ axis, we assume that the coupling constants belong to the domain $\mathcal{D}$ defined by

$$\tilde{\mathsf{J}}_z \ \leq \ \tilde{\mathsf{J}}_x, \ \tilde{\mathsf{J}}_y \ \leq 3|\tilde{\mathsf{J}}_z| \ \text{ and } \ \tilde{\mathsf{J}}_z \ \leq \ 0. \tag{24}$$

For couplings within $\mathcal{D}$, the spin wave calculations[4,5] show that the spectrum encompasses a flat mode E_o at:

$$E_o = \sqrt{(3|\tilde{\mathsf{J}}_z| - \tilde{\mathsf{J}}_x)(3|\tilde{\mathsf{J}}_z| - \tilde{\mathsf{J}}_y)} \tag{25}$$

along with dispersive branches. To determine the values compatible with our experiments, we let the three parameters vary within $\mathcal{D}$, for the fixed value of θ given above. Our goodness criterion is based on a χ^2 defined by the squared difference between the calculated and measured spin wave energies. In our definition, χ^2 takes into account $\mathbf{q}$ points along $(-2, h, 2-h)$ at $H = 0$ and 0.75 T. The inverse χ^2 is shown in Supplementary Figure 11. We find that the spin wave spectrum is well reproduced for a sub-domain $\mathcal{D}'$ of $\mathcal{D}$ with (units are in K):

$$\begin{aligned} \tilde{\mathsf{J}}_z =& -0.5 \pm 0.05 \\ 0.66 \approx& (1.5 - \tilde{\mathsf{J}}_x)(1.5 - \tilde{\mathsf{J}}_y) \end{aligned} \tag{26}$$

but one is left with a quite large uncertainty regarding $\tilde{\mathsf{J}}_x$ and $\tilde{\mathsf{J}}_y$. We note that the best χ^2 is obtained for $\tilde{\mathsf{J}}_x \sim 0.16$, $\tilde{\mathsf{J}}_y \sim 0.97$ and $\theta = 69^\circ$, yet this choice does not predict the correct neutron intensities.

To solve this problem and resolve this uncertainty, we investigate more closely the sets of parameters found in $\mathcal{D}'$. To this end, we consider, for each set of coupling constants in $\mathcal{D}'$, a trial θ such that $0 \le \theta \le \pi/2$, and compare the calculated and measured magnetic moments for the different ions in the unit cell (see Supplementary Note 1 which discusses the neutron diffraction). A new χ^2 is defined as the sum of squared differences between those quantities for a series of fields between -1 and 1 T. For:

$$\tilde{\mathsf{J}}_z = (-0.5 \pm 0.05)\ \mathrm{K},\ \tilde{\mathsf{J}}_x = (1.0 \pm 0.05)\ \mathrm{K},\ \tilde{\mathsf{J}}_y = (0.066 \pm 0.2)\ \mathrm{K}, \theta = 72 \pm 10^\circ \tag{27}$$

corresponding to

$$\mathsf{J}_x = (-0.36 \pm 0.16)\ \mathrm{K},\ \mathsf{J}_y = (0.066 \pm 0.2)\ \mathrm{K},\ \mathsf{J}_z = (0.86 \pm 0.15)\ \mathrm{K},\ \mathsf{J}_{xz} = (0.44 \pm 0.15)\ \mathrm{K} \tag{28}$$

we find a good agreement with INS results and the field induced magnetic structure. Note that the latter values seem to strongly differ from those given in Supplementary Reference 5, but this difference is essentially due to the value of θ. The dispersions along $(-2, h, 2-h)$ shown in Supplementary Figure 12(a) at $\mu_0 H = 0.25$ and 0.75 T capture the rise of the B branch, as well as the shift and the narrowing of the A branch with increasing field. Calculated constant energy maps at $H = 0$ and 0.25 T are also shown in Supplementary Figures 13 and 14. The energies are chosen to describe the flat modes and the two (A and B) dispersing branches already observed in Supplementary Figure 12. The calculations show that both of them stem from the pinch points and close at the $(-2, 2, 0)$ wavevectors, as in experiment. Importantly, the structure factor of the flat mode strongly evolves with field. At $\mu_0 H = 0.25$ T, the calculation especially demonstrates the rise of a dynamical kagome ice pinch points pattern (left upper panel of Supplementary Figure 14).

Furthermore, the calculated magnetic structure experiences a field evolution which resembles the diffraction data. Supplementary Figures 12(b) and (c) compare the calculated magnetic moment of the apical Nd^{3+} ion (y) and of the three kagome ions (x) with diffraction data, at 50 mK and 0.5 K respectively. While the overall agreement is good, there is a significant discrepancy between the experimentally observed field at which the abrupt transition occurs and the field value produced by the calculation at 50 mK. In the experiment, it is due to the transition from the all in – all out configuration to the 3 in – 1 out structure and occurs at about 0.08 T. In the calculation, a similar abrupt transition also occurs, but at about 1 T at 50 mK and 0.35 T at 0.5 K. The origin of this transition is better understood when looking at the τ_x components of the pseudo-spin. Supplementary Figure 12(d) shows that the transition occurs when the τ_x component of the apical spins changes sign, becoming positive. In other words, it corresponds to the field where the apical spins are fully aligned along the **z** axis. Because of the primarily antiferromagnetic $\tilde{\mathsf{J}}_z$ interactions, an all in – all out configuration is always favoured. Here the field already imposes a 3 in – 1 out configuration for the **z** components, which is not favourable. A gain in exchange energy can be however achieved if the **x** components keep the same sign, hence precipitating the transition.

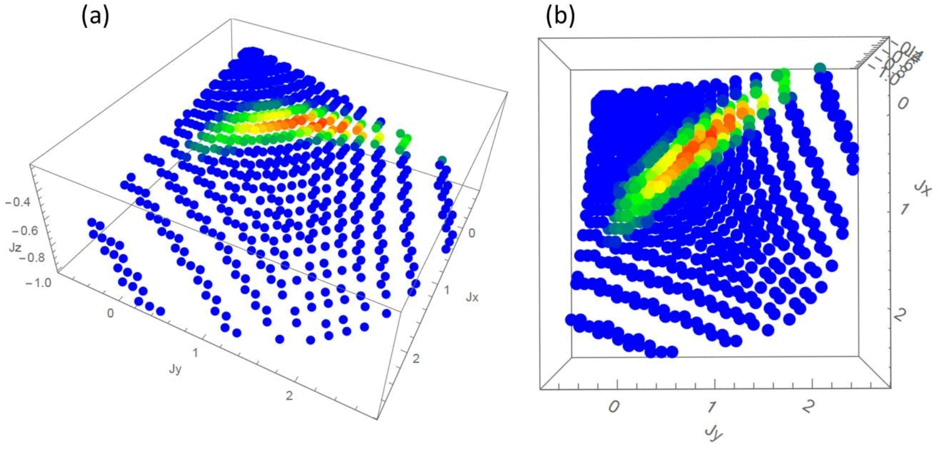

Supplementary Figure 11. **Inverse of χ^2 as a function of the Hamiltonian parameters.** The colour-map is chosen such that the best values are in red and the worst ones in blue. (a) Map as a function of the three parameters $\tilde{\mathsf{J}}_x, \tilde{\mathsf{J}}_y$ and $\tilde{\mathsf{J}}_z$. (b) Same data projected onto the $(\tilde{\mathsf{J}}_x, \tilde{\mathsf{J}}_y)$ plane.

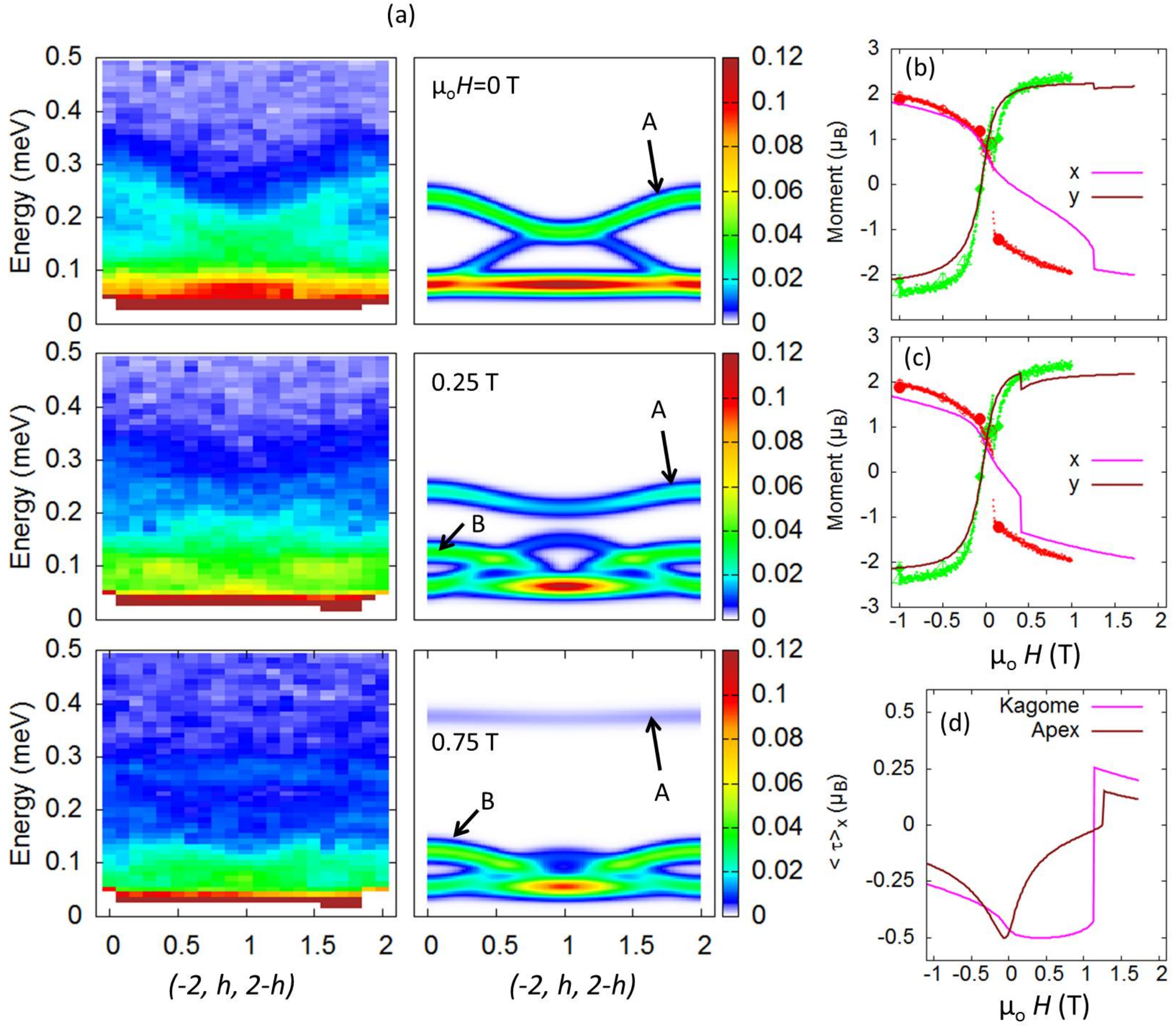

Supplementary Figure 12. **Comparison between measurements and calculations for the dispersions and the ordered moment.** (a) Measured and calculated dispersions along $(-2, h, 2-h)$ for 0, 0.25 and 0.75 T. The parameters are $\mathsf{J}_x = -0.36$ K, $\mathsf{J}_y = 0.066$ K, $\mathsf{J}_z = 0.86$ K, $\mathsf{J}_{xz} = 0.44$ K. Above 0.25 T, a new dispersive branch (labelled B) rises at low energy. The original branch (labelled A) already present in zero field narrows and shifts towards higher energy with increasing field. **(b)** and **(c)** Calculated magnetic moment of the apical Nd^{3+} ion (y) and of the three kagome ions (x) at 50 mK and 0.5 K respectively. The transition is clearly better reproduced at the effective temperature of 0.5 K. **(d)** Component τ_x of the pseudo-spin. Interestingly, it is always of the same sign for both the apical and kagome pseudo-spins. This results from the large negative value of J_z.

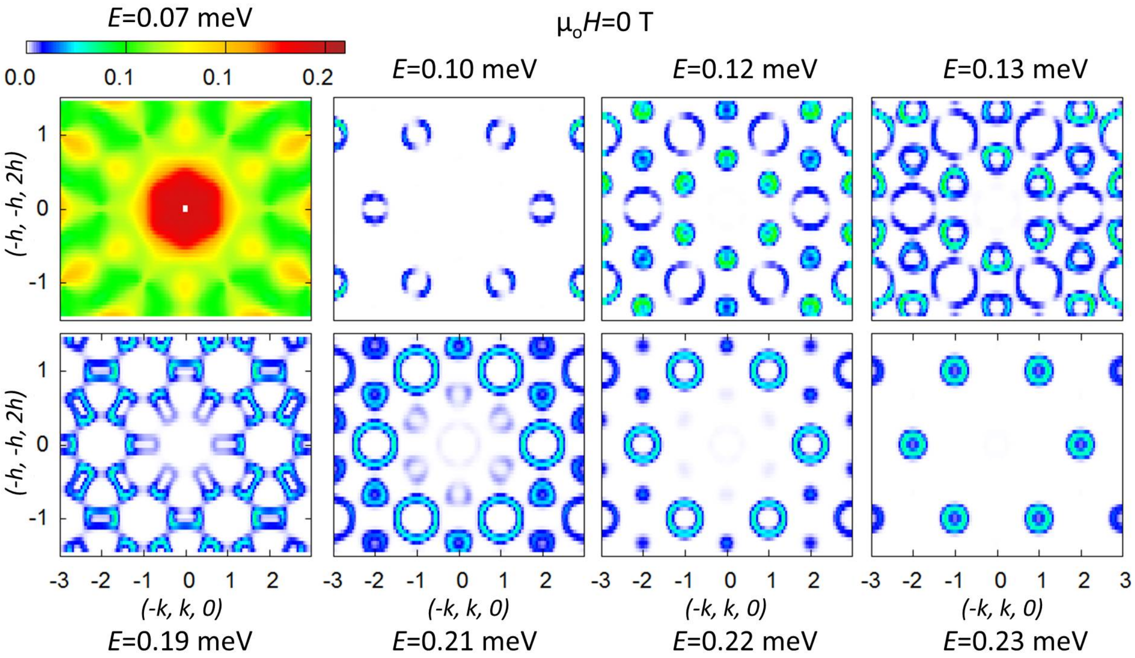

Supplementary Figure 13. **Calculated constant energy maps at zero field.** The energies are chosen to describe the flat and dispersing modes that can be also observed in Supplementary Figure 12.

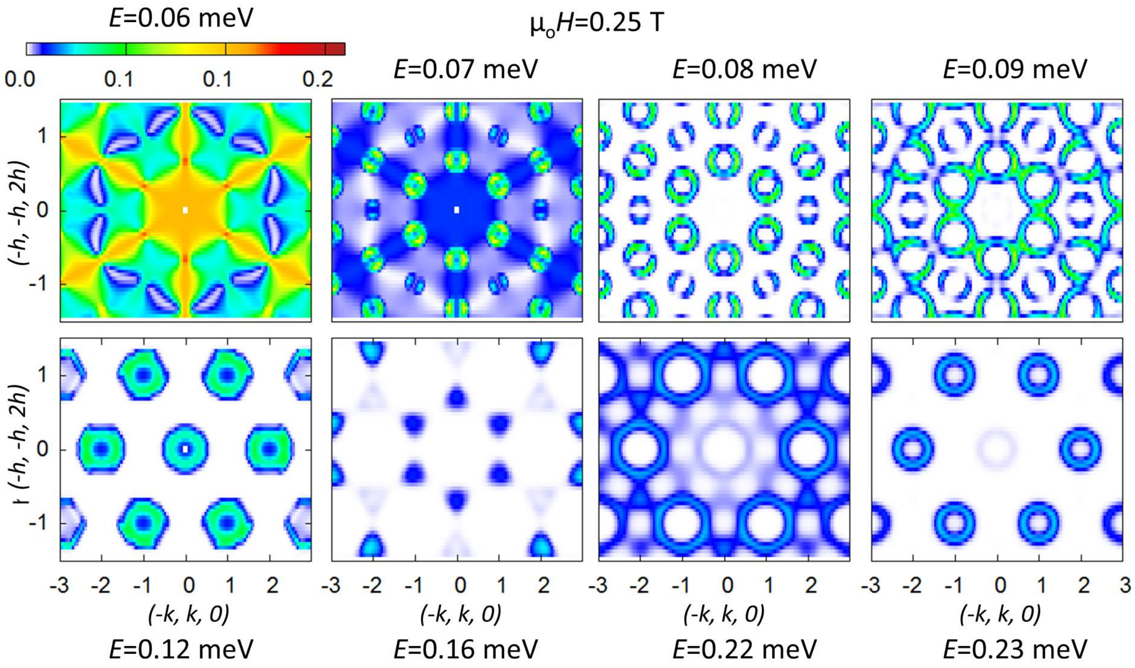

Supplementary Figure 14. **Calculated constant energy maps at 0.25 T**. The energies are chosen to describe the new dispersing modes as well as the higher energy branch. Comparing the flat mode structure factor with the one displayed in Supplementary Figure 13 clearly demonstrates the rise of pinch points in the scattering plane.

Supplementary Note 5. Decomposition of the spectrum into divergence free and divergence full dynamical fields

The decomposition of the spectrum in terms of (lattice) divergence free and divergence full dynamical fields has first been shown in Supplementary Reference 5. Here, we follow a similar route, expanding the calculations by taking into account the effect of the magnetic field. We first write the equations of motion in zero field taking advantage of the fact that the XYZ model is written using site dependent local bases:

$$\frac{d\mathbf{m}_i}{dt} = \mathbf{m}_i \times \sum_j \bar{\mathrm{J}}\ \mathbf{m}_j \tag{29}$$

where the sum over j sites is restricted to nearest neighbours and $\bar{\mathrm{J}}$ is the exchange tensor. Here, $\mathbf{m}_i$ is written in the local basis attached to site i, and not in the global Cartesian frame.

Our derivation assumes that the ground state is uniform, i.e. that the spins locally point along the same *local* direction, which is nothing but an all in – all out like configuration. In other terms, we assume here that the system is essentially an antiferromagnet. Calling $\mathbf{m}^{\mathrm{o}}$ the local equilibrium magnetization, we linearize those equations to obtain:

$$\frac{d\delta\mathbf{m}_i}{dt} = \mathbf{m}^{\mathrm{o}} \times \sum_j \bar{\mathrm{J}}\ \delta\mathbf{m}_j + \delta\mathbf{m}_i \times 6\ \bar{\mathrm{J}}\ \mathbf{m}^{\mathrm{o}}. \tag{30}$$

Note that $\mathbf{m}^{\mathrm{o}}$ is indeed identical, whatever the site, since it is written in local bases.

Since $\mathbf{m}^{\mathrm{o}}$ is an equilibrium solution, we also note that:

$$0 = \mathbf{m}^{\mathrm{o}} \times \sum_j \bar{\mathrm{J}}\ \mathbf{m}^{\mathrm{o}} \tag{31}$$

hence, $\mathbf{m}^{\mathrm{o}}$ and $\bar{\mathrm{J}}\ \mathbf{m}^{\mathrm{o}}$ must be collinear. Writing the exchange tensor in the diagonal form (see section III):

$$\bar{\mathrm{J}} = \tilde{\mathrm{J}}_x\ \tilde{\mathbf{x}}\tilde{\mathbf{x}} + \tilde{\mathrm{J}}_y\ \tilde{\mathbf{y}}\tilde{\mathbf{y}} + \tilde{\mathrm{J}}_z\ \tilde{\mathbf{z}}\tilde{\mathbf{z}} \tag{32}$$

we observe that if $\mathbf{m}^{\mathrm{o}}$ is along one of these axes, for instance $\tilde{\mathbf{z}}$, then

$$\bar{\mathrm{J}}\ \mathbf{m}^{\mathrm{o}} = \tilde{\mathrm{J}}_z\ (\tilde{\mathbf{z}} \cdot \mathbf{m}^{\mathrm{o}})\ \tilde{\mathbf{z}} = \tilde{\mathrm{J}}_z\ m^{\mathrm{o}}\ \tilde{\mathbf{z}}. \tag{33}$$

In other words, $\mathbf{m}^{\mathrm{o}}$ and $\bar{\mathrm{J}}\ \mathbf{m}^{\mathrm{o}}$ are indeed collinear. The energy is finally minimized when $\mathbf{m}^{\mathrm{o}}$ is along the axis corresponding to the smallest eigenvalue among $(\tilde{\mathrm{J}}_x, \tilde{\mathrm{J}}_y, \tilde{\mathrm{J}}_z)$.

Thanks to the pyrochlore structure, the sum over the neighbours sites j can be written in terms of the components of the generalized charge:

$$Q_{\boxtimes}^{u=x,y,z} = \sum_{i\in\boxtimes} m_i^u \tag{34}$$

defined in each tetrahedron $\boxtimes$ (see Supplementary Figure 15). Note that by definition, those charges reside on the dual lattice of the pyrochlore, which is the diamond lattice. Since the latter is bipartite, we shall consider two dual sub-lattices, A and B, with:

$$Q_{\boxtimes A}^u = +\sum_{i\in\boxtimes A} m_i^u \tag{35}$$

for A diamond sites and

$$Q_{\boxtimes B}^u = -\sum_{i\in\boxtimes B} m_i^u \tag{36}$$

for B diamond sites. Interestingly, the uniform static solution $\mathbf{m}^{\mathrm{o}}$ can be understood as a staggered pattern, from the point of view of the charge, with $\mathbf{Q}_{\boxtimes} = \pm 4\mathbf{m}^{\mathrm{o}}$. Finally, using these definitions of $\mathbf{Q}_{\boxtimes A,B}$, we shall write the equation

of motion as:

$$\begin{aligned}\frac{d\delta\mathbf{m}_i}{dt} =&\mathbf{m}^{\rm o} \times \bar{\mathtt{J}}\ \left(\mathbf{Q}_{\boxtimes_i A} - \delta\mathbf{m}_i - (\mathbf{Q}_{\boxtimes_i B} + \delta\mathbf{m}_i)\right) + \delta\mathbf{m}_i \times 6\ \bar{\mathtt{J}}\ \mathbf{m}^{\rm o} \\ =&\mathbf{m}^{\rm o} \times \bar{\mathtt{J}}\ \left(\mathbf{Q}_{\boxtimes_i A} - \mathbf{Q}_{\boxtimes_i B} - 2\delta\mathbf{m}_i\right) + \delta\mathbf{m}_i \times 6\ \bar{\mathtt{J}}\ \mathbf{m}^{\rm o}\end{aligned} \tag{37}$$

where the indexes $\boxtimes_i A$ and $\boxtimes_i B$ denote the A and B tetrahedra the site i belongs to. This form of the linearized equations of motion is the starting point to derive a peculiar partition of the solutions.

Charged solutions: We start with the subset of charged solutions. Forming the equations of motion for $\mathbf{Q}_{\boxtimes A,B}$ using the previous equations, one obtains:

$$\begin{aligned}\frac{d\mathbf{Q}_{\boxtimes A}}{dt} =&\mathbf{m}^{\rm o} \times \bar{\mathtt{J}}\ \left(4\ \mathbf{Q}_{\boxtimes A} - \sum_{\boxtimes B}\mathbf{Q}_{\boxtimes B} - 2\ \mathbf{Q}_{\boxtimes A}\right) + \mathbf{Q}_{\boxtimes A} \times 6\ \bar{\mathtt{J}}\ \mathbf{m}^{\rm o} \\ =&\mathbf{Q}_{\boxtimes A} \times 6\ \bar{\mathtt{J}}\ \mathbf{m}^{\rm o} + \mathbf{m}^{\rm o} \times 2\ \bar{\mathtt{J}}\ \mathbf{Q}_{\boxtimes A} - \sum_{\boxtimes B}\mathbf{m}^{\rm o} \times \bar{\mathtt{J}}\ \mathbf{Q}_{\boxtimes B}.\end{aligned} \tag{38}$$

Those solutions correspond to the propagation of the charge throughout the diamond lattice. We shall also make several points:

- the $A \leftrightarrow B$ symmetry is clear from the above equation.
- The sum over $\boxtimes B$ refers to the four neighbouring B tetrahedra of a given A tetrahedron.
- Since for a lattice containing N site, there are $(N/4 + N/4)$ A and B tetrahedra, the number of these modes is $N/2$. In the Helmholtz-Hodge decomposition of the $\mathbf{m}_i$ field, these modes are obviously associated with the dynamical divergence full field.
- Finally, we write the exchange tensor in its diagonal form $\bar{\mathtt{J}} = \tilde{\mathtt{J}}_x\ \tilde{\mathbf{x}}\tilde{\mathbf{x}} + \tilde{\mathtt{J}}_y\ \tilde{\mathbf{y}}\tilde{\mathbf{y}} + \tilde{\mathtt{J}}_z\ \tilde{\mathbf{z}}\tilde{\mathbf{z}}$ (assuming that $\mathbf{m}^{\rm o}$ is along $\tilde{\mathbf{z}}$, while $(\tilde{\mathbf{x}}, \tilde{\mathbf{y}})$ span the plane perpendicular to $\mathbf{m}^{\rm o}$) and introduce the coordinates of the charge in the corresponding $(\tilde{\mathbf{x}}, \tilde{\mathbf{y}}, \tilde{\mathbf{z}})$ frame:

$$\mathbf{Q}_{\boxtimes A} = \begin{pmatrix} Q^{\tilde{x}}_{\boxtimes A} \\ Q^{\tilde{y}}_{\boxtimes A} \\ Q^{\tilde{z}}_{\boxtimes A}\end{pmatrix} \tag{39}$$

The $Q^{\tilde{z}}_{\boxtimes A}$ components remain constant (consistent with the all in – all out structure), while the $Q^{\tilde{x}}_{\boxtimes A}$ and $Q^{\tilde{y}}_{\boxtimes A}$ components evolve in time following:

$$\frac{d\mathbf{Q}_{\boxtimes A}}{dt} = \begin{pmatrix} 0 & 6\tilde{\mathtt{J}}_z m^{\rm o} - 2\tilde{\mathtt{J}}_y m^{\rm o} \\ -(6\tilde{\mathtt{J}}_z m^{\rm o} - 2\tilde{\mathtt{J}}_x m^{\rm o}) & 0 \end{pmatrix} \mathbf{Q}_{\boxtimes A}\ -\ \sum_{\boxtimes B}\begin{pmatrix} 0 & \tilde{\mathtt{J}}_y m^{\rm o} \\ -\tilde{\mathtt{J}}_x m^{\rm o} & 0\end{pmatrix}\ \mathbf{Q}_{\boxtimes B}. \tag{40}$$

Alternate loops weathervane modes: The second set of solutions is constructed by introducing loops $\mathcal{L}$ consisting of N sites in the pyrochlore lattice. Note that the shortest of these loops are the hexagons that form the kagome planes. We consider:

$$\mathbf{A} = \sum_{k\in\mathcal{L}} u_k \delta\mathbf{m}_k. \tag{41}$$

Forming the equation of motion for $\mathbf{A}$, we obtain:

$$\begin{aligned}\frac{d\mathbf{A}}{dt} =&\mathbf{A} \times 6\ \bar{\mathtt{J}}\ \mathbf{m}^{\rm o} - \mathbf{m}^{\rm o} \times 2\ \bar{\mathtt{J}}\ \mathbf{A} \\ &+ \mathbf{m}^{\rm o} \times \bar{\mathtt{J}}\ \left(u_1\mathbf{Q}_{\boxtimes_1 A} - u_1\mathbf{Q}_{\boxtimes_1 B} + ... + u_N\mathbf{Q}_{\boxtimes_N A} - u_N\mathbf{Q}_{\boxtimes_N B}\right).\end{aligned} \tag{42}$$

We note that two successive sites belong, for example, to the same A tetrahedron but to different B ones: $\boxtimes_1 A = \boxtimes_2 A$, $\boxtimes_3\ A = \boxtimes_4 A$, $... = ...$, $\boxtimes_{N-1}\ A = \boxtimes_N A$ and $\boxtimes_2 B = \boxtimes_3 B$, $\boxtimes_4\ B = \boxtimes_5 B$, $... = ...$

Furthermore, since $\mathcal{L}$ is a loop, we have $\boxtimes_N B = \boxtimes_1 B$, hence:

$$\begin{aligned}\sum_{k=1,N} u_k \mathbf{Q}_{\boxtimes_k A} =&(u_1+u_2)\mathbf{Q}_{\boxtimes_1 A} + (u_3+u_4)\mathbf{Q}_{\boxtimes_3 A} + ...\\ \sum_{k=1,N} u_k \mathbf{Q}_{\boxtimes_k B} =&(u_N+u_1)\mathbf{Q}_{\boxtimes_1 B} + (u_2+u_3)\mathbf{Q}_{\boxtimes_1 B} + ...\end{aligned} \tag{43}$$

Assuming alternate coefficients $u_{k+1} = -u_k$, and $u_N = -u_1$, we obtain:

$$\frac{d\mathbf{A}}{dt} = \mathbf{A} \times 6\ \bar{\mathrm{J}}\ \mathbf{m}^o - \mathbf{m}^o \times 2\ \bar{\mathrm{J}}\ \mathbf{A} \tag{44}$$

This shows that the alternate loop solutions correspond to a resonating dispersionless field, decoupled from the charged solutions. To determine the corresponding energy, we write the exchange tensor in its diagonal form: $\bar{\mathrm{J}} = \tilde{\mathrm{J}}_x\ \tilde{\mathbf{x}}\tilde{\mathbf{x}} + \tilde{\mathrm{J}}_y\ \tilde{\mathbf{y}}\tilde{\mathbf{y}} + \tilde{\mathrm{J}}_z\ \tilde{\mathbf{z}}\tilde{\mathbf{z}}$, and assume that $\tilde{\mathbf{z}}$ is along $\mathbf{m}^o$. We thus have:

$$\begin{aligned}\frac{d\mathbf{A}}{dt} =&6\tilde{\mathrm{J}}_z m^o (A_{\tilde{x}}\tilde{\mathbf{x}} + A_{\tilde{y}}\tilde{\mathbf{y}}) \times \tilde{\mathbf{z}} - 2m^o \tilde{\mathbf{z}} \times (\tilde{\mathrm{J}}_x \tilde{\mathbf{x}} A_{\tilde{x}} + \tilde{\mathrm{J}}_y \tilde{\mathbf{y}} A_{\tilde{y}} + \tilde{\mathrm{J}}_z \tilde{\mathbf{z}} A_{\tilde{z}})\\ =&6\tilde{\mathrm{J}}_z m^o (-A_{\tilde{x}}\tilde{\mathbf{y}} + A_{\tilde{y}}\tilde{\mathbf{x}}) - 2m^o(\tilde{\mathrm{J}}_x \tilde{\mathbf{y}} A_{\tilde{x}} - \tilde{\mathrm{J}}_y \tilde{\mathbf{x}} A_{\tilde{y}})\\ =&\begin{pmatrix} 0 & 6\tilde{\mathrm{J}}_z m^o + 2\tilde{\mathrm{J}}_y m^o \\ -(6\tilde{\mathrm{J}}_z m^o + 2\tilde{\mathrm{J}}_x m^o) & 0 \end{pmatrix} \mathbf{A}\end{aligned} \tag{45}$$

The eigenvalues of above matrix are of the form $\pm i\Delta$ with:

$$\Delta = 2m^o\ \sqrt{(3\tilde{\mathrm{J}}_z + \tilde{\mathrm{J}}_x)(3\tilde{\mathrm{J}}_z + \tilde{\mathrm{J}}_y)} \tag{46}$$

and Δ corresponds to the frequency of those dispersionless modes. Since we consider only the cases where $\tilde{\mathrm{J}}_z < 0$, we write $\tilde{\mathrm{J}}_z = -|\tilde{\mathrm{J}}_z|$ and finally:

$$\Delta = 2m^o\ \sqrt{(3|\tilde{\mathrm{J}}_z| - \tilde{\mathrm{J}}_x)(3|\tilde{\mathrm{J}}_z| - \tilde{\mathrm{J}}_y)} \tag{47}$$

which is the energy of the flat mode described in Supplementary notes 3 and 4.

Influence of a [111] **magnetic field:** Applying a magnetic field along one of the $\langle 111 \rangle$ high symmetry directions is especially interesting as the sites of the pyrochlore lattice separate in kagome (K) and triangular (T) planes forming the subset of apical spins. The latter are rapidly polarized by the field since the Ising direction is precisely along $\mathbf{H}$. The equations of motion can now be written as:

$$\frac{d\mathbf{m}_i}{dt} = \mathbf{m}_i \times \left(\sum_j \bar{\mathrm{J}}\ \mathbf{m}_j + g_i \mathbf{H} \right) \tag{48}$$

and we still consider a linearized version around a uniform solution. However, as emphasized above, the field has different influences depending on the site: for the apical sites, $g_i\mathbf{H} = -g_{\parallel} H\ \mathbf{z}$ while for the kagome sites, $g_i\mathbf{H} = g_{\parallel} H/3\ \mathbf{z}$. As a result, we shall consider a generalized solution $\mathbf{m}^{o,(K,T)}$ with different equilibrium values for the apical and kagome sites:

$$\begin{aligned}0 =&\mathbf{m}^{o,T} \times \left(6\ \bar{\mathrm{J}}\ \mathbf{m}^{o,K} - g_{\parallel} H\ \mathbf{z}\right)\\ 0 =&\mathbf{m}^{o,K} \times \left(4\ \bar{\mathrm{J}}\ \mathbf{m}^{o,K} + 2\ \bar{\mathrm{J}}\ \mathbf{m}^{o,T} + g_{\parallel} H/3\ \mathbf{z}\right).\end{aligned} \tag{49}$$

Our aim is to show that once the apical spins are fully polarized, the kagome ice displays, on one hand charged solutions, and on the other, flat alternate loop modes. Owing to the dimensional reduction, the charges are, however, kagome charges, and the loops are confined within the kagome layers. To this end, we introduce the kagome charge $\mathbf{Q}_{\triangle}^{u=x,y,z} = \sum_{i\in\triangle} m_i^u$ of a given kagome triangle $\triangle$. It is worth noting that the same bipartite property holds in this 2D case. As a result, A and B triangles can be defined, in close analogy with the pyrochlore case (see Supplementary Figure 16). Let us consider, on one hand, a site i where the i index runs in the kagome plane. This site belongs to an A and to a B kagome triangle, and has two neighbours sites in the T triangular planes, one in the top ($j = t_i$) plane

and one in the bottom ($j = b_i$) plane. The equation of motion can be written as:

$$\frac{d\delta\mathbf{m}_i^K}{dt} = \mathbf{m}^{o,K} \times \bar{\mathsf{J}} \left(\mathbf{Q}_{\triangle_i A} - \delta\mathbf{m}_i^K - \left(\mathbf{Q}_{\triangle_i B} + \delta\mathbf{m}_i^K\right) + \sum_{j=t_i,b_i} \bar{\mathsf{J}}\ \delta\mathbf{m}_j^T \right) + \delta\mathbf{m}_i^K \times \left(4\bar{\mathsf{J}}\ \mathbf{m}^{o,K} + 2\bar{\mathsf{J}}\ \mathbf{m}^{o,T} + g_{\parallel} H/3\ \mathbf{z}\right). \tag{50}$$

On the other hand, let's consider a site j where j runs in the triangular plane. This site is also the apical site of A and B kagome triangles, denoted $\triangle_j A$ and $\triangle_i B$. With this notation, the equation of motion becomes:

$$\frac{d\delta\mathbf{m}_j^T}{dt} = \mathbf{m}^{o,T} \times \bar{\mathsf{J}}\ \left(\mathbf{Q}_{\triangle_j A} - \mathbf{Q}_{\triangle_j B}\right) + \delta\mathbf{m}_j^T \times 6\ \bar{\mathsf{J}}\ \mathbf{m}^{o,K} - g_{\parallel} H\ \delta\mathbf{m}_j^T \times \mathbf{z}. \tag{51}$$

If the field is strong enough to fully polarize the apical sites, we may neglect the term $\sum_{j=t_i,b_i} \bar{\mathsf{J}}\ \delta\mathbf{m}_j^T$, and hence we obtain:

$$\frac{d\mathbf{m}_i^K}{dt} = \mathbf{m}^{o,K} \times \bar{\mathsf{J}} \left(\mathbf{Q}_{\triangle_i A} - \mathbf{Q}_{\triangle_i B} - 2\delta\mathbf{m}_i^K\right) + \delta\mathbf{m}_i^K \times \left(4\bar{\mathsf{J}}\ \mathbf{m}^{o,K} + 2\bar{\mathsf{J}}\ \mathbf{m}^{o,T} + g_{\parallel} H/3\ \mathbf{z}\right). \tag{52}$$

In this limit, we obtain a decomposition of the excitations similar to what we found in zero field, in terms of divergence full and divergence free fields, but within the kagome layers. The charged solutions are given by:

$$\frac{d\mathbf{Q}_{\triangle A}}{dt} = \mathbf{Q}_{\triangle A} \times \left(4\bar{\mathsf{J}}\ \mathbf{m}^{o,K} + 2\bar{\mathsf{J}}\ \mathbf{m}^{o,T} + g_{\parallel} H/3\ \mathbf{z}\right) + \mathbf{m}^{o,K} \times \bar{\mathsf{J}}\ \mathbf{Q}_{\triangle A} - \sum_{\triangle B} \mathbf{m}^{o,K} \times \bar{\mathsf{J}}\ \mathbf{Q}_{\triangle B} \tag{53}$$

and the dispersionless solutions by hexagonal loops of alternate spins:

$$\frac{d\mathbf{A}}{dt} = \mathbf{A} \times \left(4\bar{\mathsf{J}}\ \mathbf{m}^{o,K} + 2\bar{\mathsf{J}}\ \mathbf{m}^{o,T} + g_{\parallel} H/3\ \mathbf{z}\right) - \ \mathbf{m}^{o,K} \times \bar{\mathsf{J}}\ \mathbf{A}. \tag{54}$$

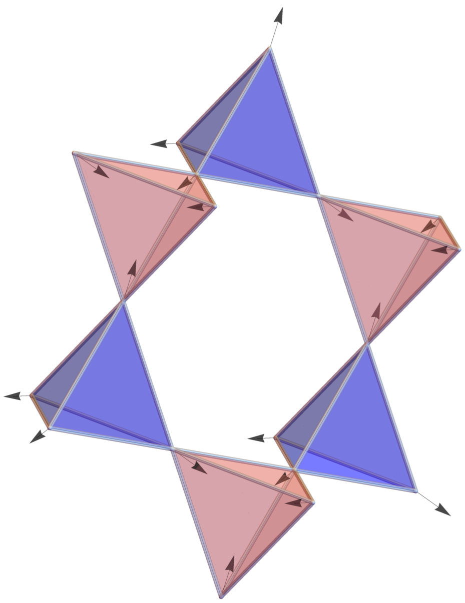

Supplementary Figure 15. **Partial view of the pyrochlore lattice in the all in − all out magnetic structure depicted by the black arrows**. The staggered charged pattern is shown using blue and red tetrahedra. The nodes of the diamond lattice, dual of the pyrochlore, are the centres of the tetrahedra. This dual lattice hosts the charge. It is bipartite, which allows one to distinguish A and B charges. Clearly, each pyrochlore site belongs to an A and to a B tetrahedron.

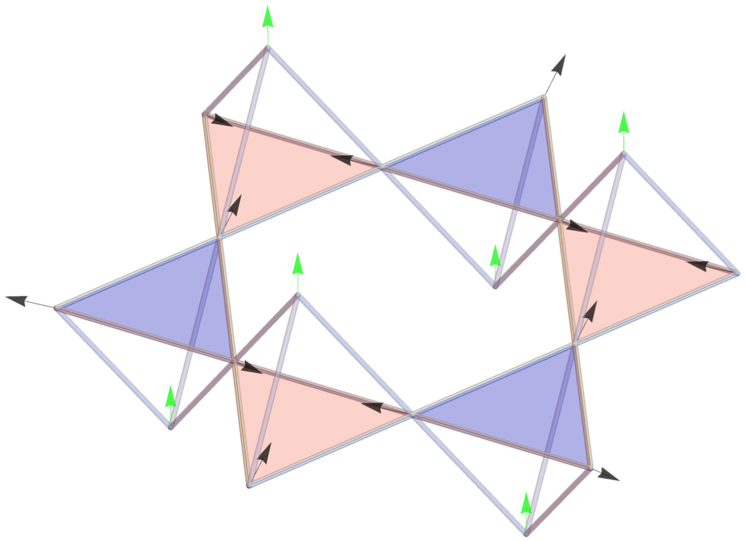

Supplementary Figure 16. **Partial view of the pyrochlore lattice in the 3 in − 1 out magnetic structure**. The black and the green arrows depict the kagome and apical spins respectively. The staggered charged pattern corresponding to a uniform solution is shown using blue and red triangles.

Supplementary References

1. Rodríguez-Carvajal, J. Recent advances in magnetic structure determination by neutron powder diffraction. *Physica B* **192**, 55 (1993). http://www.ill.eu/sites/fullprof/
2. Ewings, R. A., Buts, A., Le, M. D., van Duijn, J., Bustinduy, I., and Perring, T. G. , *Nucl. Instrum. Methods Phys. Res., Sect. A* **834**, 132 (2016). See also Perring, T. G., et al. horace.isis.rl.ac.uk/MainPage.
3. Huang, Y.-P., Chen, G. and Hermele, M. Quantum spin ices and topological phases from dipolar-octupolar doublets on the pyrochlore lattice. *Phys. Rev. Lett.* **112**, 167203 (2014).
4. Petit, S., Lhotel, E., Canals, B., Ciomaga Hatnean, M., Ollivier, J., Muttka, H., Ressouche, E., Wildes, A. R., Lees, M. R., and Balakrishnan, G. Observation of magnetic fragmentation in spin ice, *Nat. Phys.* **12**, 746-750 (2016).
5. Benton, O. Quantum origins of moment fragmentation in $Nd_2Zr_2O_7$. *Phys. Rev. B* **94**, 104430 (2016).
6. Lhotel, E., Petit, S., Guitteny, S., Florea, O., Ciomaga Hatnean, M., Colin, C., Ressouche, E., Lees, M. R., and Balakrishnan, G. Fluctuations and all-in−all-out ordering in dipole-octopole $Nd_2Zr_2O_7$. *Phys. Rev. Lett.* **115**, 197202 (2015).
